# Chromatin accessibility: a window into the genome

**DOI:** 10.1186/1756-8935-7-33

**Published:** 2014-11-20

**Authors:** Maria Tsompana, Michael J Buck

**Affiliations:** New York State Center of Excellence in Bioinformatics and Life Sciences, State University of New York at Buffalo, 701 Ellicott St, Buffalo, NY 14203 USA; Department of Biochemistry, State University of New York at Buffalo, Buffalo, NY USA

**Keywords:** Chromatin, MNase, DNase, ATAC, FAIRE, Sequencing, Library, Epigenome, Histone, Nucleosome

## Abstract

Transcriptional activation throughout the eukaryotic lineage has been tightly linked with disruption of nucleosome organization at promoters, enhancers, silencers, insulators and locus control regions due to transcription factor binding. Regulatory DNA thus coincides with open or accessible genomic sites of remodeled chromatin. Current chromatin accessibility assays are used to separate the genome by enzymatic or chemical means and isolate either the accessible or protected locations. The isolated DNA is then quantified using a next-generation sequencing platform. Wide application of these assays has recently focused on the identification of the instrumental epigenetic changes responsible for differential gene expression, cell proliferation, functional diversification and disease development. Here we discuss the limitations and advantages of current genome-wide chromatin accessibility assays with especial attention on experimental precautions and sequence data analysis. We conclude with our perspective on future improvements necessary for moving the field of chromatin profiling forward.

**Electronic supplementary material:**

The online version of this article (doi:10.1186/1756-8935-7-33) contains supplementary material, which is available to authorized users.

## Introduction: chromatin accessibility

Eukaryotic chromatin is tightly packaged into an array of nucleosomes, each consisting of a histone octamer core wrapped around by 147 bp of DNA and separated by linker DNA [1–3]. The nucleosomal core consists of four histone proteins [1] that can be post-translationally altered by at least 80 known covalent modifications [4, 5] or replaced by histone variants [6–8]. Positioning of nucleosomes throughout a genome has a significant regulatory function by modifying the *in vivo* availability of binding sites to transcription factors (TFs) and the general transcription machinery and thus affecting DNA-dependent processes such as transcription, DNA repair, replication and recombination [9]. Experiments designed to decipher how nucleosome positioning regulates gene expression have led to the understanding that transcriptional activation coincides with nucleosome perturbation, whereas transcriptional regulation requires the repositioning of nucleosomes throughout the eukaryotic lineage [10–18].

Nucleosome eviction or destabilization at promoters and enhancers results from the binding of specific regulatory factors responsible for transcriptional activation in eukaryotes [19, 20]. Open or accessible regions of the genome are, thus, regarded as the primary positions for regulatory elements [21] and have been historically characterized by nuclease hypersensitivity *in vivo*[22]. Notably, changes in chromatin structure have been implicated with many aspects of human health, as a result of mutations in chromatin remodelers that affect nucleosome positioning [23–25]. Therefore, current interest is placed on collecting and comparing genome-wide chromatin accessibility, to locate instrumental epigenetic changes that accompany cell differentiation, environmental signaling and disease development. Large collaborative projects such as ENCODE [26] have become part of this major effort.

Low-throughput experiments in *Drosophila* using DNase I and MNase treatment, provided the first demonstration that active chromatin coincides with nuclease hypersensitivity, that is chromatin accessibility [27–30]. Currently, all chromatin accessibility assays separate the genome by enzymatic or chemical means and isolate either the accessible or protected locations. Isolated DNA is then quantified using a next-generation sequencing (NGS) platform. In this review, we focus on the latest methods for identifying chromatin accessibility genome-wide, and discuss the considerations for experimental design and data analysis. We conclude with current limitations that need to be overcome for this field to move forward.

## Review

### Assays for genome-wide chromatin accessibility

#### General considerations

Chromatin accessibility approaches measure *directly* the effect of chromatin structure modifications on gene transcription, in contrast to histone chromatin immunoprecipitation with NGS (ChIP-seq) (for a thorough review on ChIP-seq read [31–33]) where such effects must be inferred by presence or absence of overlapping histone tail modifications. Also, chromatin accessibility assays do not require antibodies or epitope tags that can introduce potential bias. An important limitation with all chromatin accessibility experiments is the lack of a standard for the number of replicates required to achieve accurate and reproducible results. This is because replicate number depends on the achieved signal-to-noise ratio, which can vary depending on the assay used, the assay conditions, and the cell or tissue type. In addition, replicate number is a function of technical variance, which is also experiment-specific and difficult to model in a generalized format. Following we discuss chromatin accessibility assays that *directly* (DNase-seq, FAIRE-seq and ATAC-seq) isolate accessible locations of a genome separate from MNase-seq, which *indirectly* evaluates chromatin accessibility, and present their principal mode of action, examples of application and main experimental considerations (Table 1).

**Table 1:** **Current genome-wide high-throughput chromatin accessibility assays**

	Cell type/Number	Sequencing type	Traditional approach	Genomic target	Experimental considerations	Key references
**MNase-seq**	Any cell type 1 to 10 million cells	Paired-end or Single-end	MNase digests unprotected DNA	Maps the total nucleosome population in a qualitative and quantitative manner	1. Requires many cells.	[37, 46, 49]
					2. Laborious enzyme titrations.	
					3. Probes total nucleosomal population, not active regulatory regions only.	
					4. Degrades active regulatory regions, making their detection possible only *indirectly*.	
					5. Requires 150 to 200 million reads for standard accessibility studies of the human genome.	
**DNase-seq**	Any cell type 1 to 10 million cells	Paired-end or Single-end	DNase I cuts within unprotected DNA	Maps open chromatin	1. Requires many cells.	[61, 75, 76]
					2. Time-consuming and complicated sample preparations.	
					3. Laborious enzyme titrations.	
					4. Requires 20 to 50 million reads for standard accessibility studies of the human genome.	
**FAIRE-seq**	Any cell type 100,000 to 10 million cells	Paired-end or Single-end	Based on the phenol-chloroform separation of nucleosome-bound and free sonicated areas of a genome, in the interphase and aqueous phase respectively	Maps open chromatin	1. Low signal-to-noise ratio, making computational data interpretation very difficult.	[86–90]
					2. Results depend highly on fixation efficiency.	
					3. Requires 20 to 50 million reads for standard accessibility studies of the human genome.	
**ATAC-seq**	500 to 50,000 freshly isolated cells	Paired-end	Unfixed nuclei are tagged *in vitro* with adapters for NGS by purified Tn5 transposase. Adapters are integrated into regions of accessible chromatin	Maps open chromatin, TF and nucleosome occupancy	1. Contamination of generated data with mitochondrial DNA.	[103]
					2. Immature data analysis tools.	
					3. Requires 60 to 100 million reads for standard accessibility studies of the human genome.	

ATAC: assay for transposase-accessible chromatin; DNase I: deoxyribonuclease I; FAIRE: formaldehyde-assisted isolation of regulatory elements; MNase: micrococcal nuclease.

#### MNase-seq: an indirect chromatin accessibility assay

**Figure 1:**
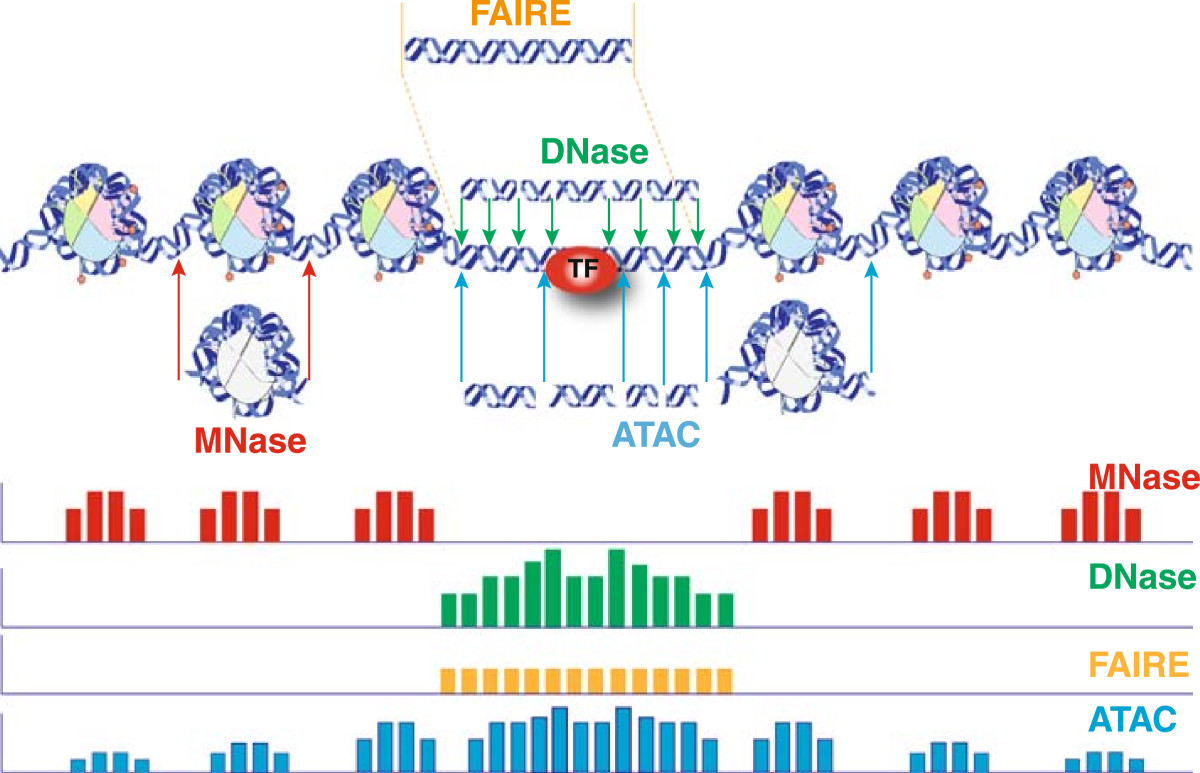
**Schematic diagram of current chromatin accessibility assays performed with typical experimental conditions.** Representative DNA fragments generated by each assay are shown, with end locations within chromatin defined by colored arrows. Bar diagrams represent data signal obtained from each assay across the entire region. The footprint created by a transcription factor (TF) is shown for ATAC-seq and DNase-seq experiments.

MNase is commonly reported as a single-strand-specific endo-exonuclease, although its exonuclease activity appears to be limited to only a few nucleotides on a single strand before cleavage of the antiparallel strand occurs [34–36]. Since the early 1970s MNase digestion has been applied to study chromatin structure in a low-throughput manner [37–40] and later in combination with tiled microarrays [41–44]. Currently, MNase digestion is used with NGS (MNase-seq or MAINE-seq [45]) for genome-wide characterization of average nucleosome occupancy and positioning in a qualitative and quantitative manner. In a typical MNase-seq experiment, mononucleosomes are extracted by extensive MNase treatment of chromatin that has been crosslinked with formaldehyde (Figure 1) [46]. The nucleosomal population is subsequently submitted to single-end (identifies one end of template) or paired-end (identifies both ends of template) NGS with a varying level of coverage depending on the exact goal of the experiment [31].

MNase-seq thus probes chromatin accessibility *indirectly* by unveiling the areas of the genome occupied by nucleosomes and other regulatory factors. Commonly referred to as a nucleosome occupancy assay, it shares same principal mode of action (enzymatic cleavage) and can provide information on TF occupancy as other chromatin accessibility assays. MNase-seq has been implemented in a number of organisms, ranging from yeast to humans, for the mapping of chromatin structure [47–49]. In addition, MNase digestion has been successfully combined with ChIP-seq for enrichment of regulatory factors or histone-tail modifications and variants. Henikoff *et al*. [50] have also introduced a modified MNase-seq protocol for library preparation of fragments down to 25 bp, allowing the mapping of both nucleosomes and non-histone proteins with high resolution.

Important considerations in the design of MNase-seq experiments include extent of chromatin crosslinking and level of digestion. Traditionally, chromatin accessibility experiments have been conducted with formaldehyde as a crosslinking agent to capture *in vivo* protein-nucleic acid and protein-protein interactions [51]. It has been observed that in the absence of crosslinking, nucleosome organization can change during regular chromatin preparation steps and thus use of formaldehyde is recommended for accurate characterization of chromatin structure [31]. Also, MNase has been shown to have a high degree of AT-cleavage specificity in limiting enzyme concentrations [52–54] and comparisons between different experiments will vary for technical reasons unless MNase digestion conditions are tightly controlled [55–57]. MNase titration experiments specifically support differential digestion susceptibility of certain nucleosome classes, with nucleosomes within promoter and ‘nucleosome-free’ regions being highly sensitive [50, 58, 59]. Thus, it has been suggested that combination of templates from different levels of MNase digestion may alleviate biased sampling of mononucleosome populations [58].

However, the cause of differences in MNase-seq output across differential levels of enzymatic digestion is difficult to assess due to the effect of inter-nucleosomal linker length on the recovered signal [55]. MNase digestion simulation experiments have provided evidence that nucleosome configurations with or near long linkers are sampled easier compared to nucleosomes with normal linkers at low levels of MNase digestion and this sampling bias dissipitates with increased levels of enzymatic cleavage (80 or 100% monos) [55]. Comparison of *in vivo* experimental data of two distinct nucleosome configurations from different MNase-seq technical preparations supports the same conclusions, and underscores the importance of standardized collection of mononucleosomes for accurate and reproducible comparisons [55]. Specifically, extensive (approximately 95 to 100% mononucleosomes) digestion of a standardized initial amount of crosslinked chromatin is considered ideal for comparisons of different MNase-seq experiments, since at that level of digestion all linkers are cut and the recovered signal is not confounded by nucleosome configuration [31, 55].

Overall, MNase-seq is a superior method for probing genome-wide nucleosome distributions and also provides an accurate way for assessing TF occupancy in a range of cell types [60]. However, it requires a large number of cells and careful enzymatic titrations for accurate and reproducible evaluation of differential substrates.

#### Direct chromatin accessibility assays

##### DNase-seq

Historically, open chromatin has been identified by the hypersensitivity of genomic sites to nuclease treatment with MNase and the non-specific double-strand endonuclease DNase I [61]. In a typical experiment, low concentrations of DNase I liberate accessible chromatin by preferentially cutting within nucleosome-free genomic regions characterized as DNase I hypersensitive sites (DHSs) (Figure 1). Early low-throughput experiments, provided the first demonstration that active genes have an altered chromatin conformation that makes them susceptible to digestion with DNase I [61]. Further research in *Drosophila* and other eukaryotes, supported the conserved observation that chromatin structure is disrupted during gene activation and that DHSs are the primary sites of active chromatin rendering access of *trans*-factors to regulatory elements [14, 27, 28, 62–65]. It has later been shown that DHSs result during gene activation [17], due to loss or temporal destabilization of one or more nucleosomes from *cis*-regulatory elements with the combinatorial action of ATP-dependent nucleosome- and histone-remodelers [20, 66, 67].

Traditionally, identification of DHSs has been based on Southern blotting with indirect end-labeling [28] and involves laborious and time-consuming steps that limit the applicability of the method to a narrow extent of the genome. Further attempts to improve the efficiency and resolution of the method have used low-throughput sequencing, real-time PCR strategies and later hybridization to tiled microarrays [68–74]. The advent of NGS gave rise to DNase-seq allowing the genome-wide identification of DHSs with unparalleled specificity, throughput and sensitivity in a single reaction. In recent times the drop of sequencing costs and the increased quality of the data have made DNase-seq the ‘golden standard’, for probing chromatin accessibility. During a typical DNase-seq experiment, isolated nuclei are submitted to mild DNase I digestion according to the Crawford or Stamatoyannopoulos protocol [75, 76]. In the Crawford protocol, DNase I digested DNA is embedded into low-melt gel agarose plugs to prevent further shearing. Optimal digestions are selected by agarose pulsed field gel electrophoresis, with an optimal smear range from 1 MB to 20 to 100 KB, and are blunt-end ligated to a biotinylated linker. After secondary enzymatic digestion with MmeI, ligation of a second biotinylated linker and library amplification, the digested population is assayed using NGS [75]. In the Stamatoyannopoulos protocol, DNA from nuclei is digested with limiting DNase I concentrations and assessed by q-PCR and/or agarose gel electrophoresis. Optimal digestions are purified with size selection of fragments smaller than 500 bp using sucrose gradients, and are submitted for high-throughput sequencing after library construction [76]. The main difference between the two protocols is that the first one depends on the single enzymatic cleavage of chromatin, whereas the latter requires double cleavage events in close proximity to each other. The Stamatoyannopoulos protocol has been preferentially used by the ENCODE consortium.

DNase-seq has been extensively used by the ENCODE consortium [26] and others to unveil cell-specific chromatin accessibility and its relation to differential gene expression in various cell lines [21, 77–79]. It has also been modified to study rotational positioning of individual nucleosomes [80] based on the inherent preference of DNase I to cut within the minor groove of DNA at approximately every ten bp around nucleosomes [79, 81, 82]. In addition, binding of sequence-specific regulatory factors within DHSs can affect the intensity of DNase I cleavage and generate footprints (digital genomic footprinting (DGF) or DNase I footprinting) that have been used to study TF occupancy at nucleotide resolution in a qualitative and quantitative manner [83]. DGF with deep sequencing has been implemented to uncover cell-specific TF binding motifs in humans, yielding expansive knowledge on regulatory circuits and the role of TF binding in relation to chromatin structure, gene expression, and cellular differentiation [19, 78]. Due to its high resolution, DGF has also allowed the probing of functional allele-specific signatures within DHSs [78].

The main controversy over DNase-seq is the ability for DNase I to introduce cleavage bias [31, 79, 81, 82, 84], thus affecting its use as a reliable TF footprint detection assay. Two recent publications clearly demonstrate that cleavage signatures traditionally attributed to protein protection of underlying nucleotides, are detected even in the absence of TF binding as a result of DNase I inherent sequence preferences that span over more than two orders of magnitude [84, 85]. This observation is strongly supported by frequent lack of correspondence between TF binding events detected with ChIP-seq versus DGF [85]. Also, TFs with transient DNA binding times in living cells leave minimal to no detectable footprints at their sites of recognition, making the quality of footprinting highly factor-dependent [84, 85]. Collectively, these findings challenge previous DGF research on TF footprinting and its applicability as a reliable recognition assay of complex factor-chromatin interactions in a dynamic timescale.

Less concerning limitations of DNase-seq are that it requires many cells and involves many sample preparation and enzyme titration steps. Success of this assay depends on the quality of nuclei preparations and small-scale preliminary experiments are essential to ascertain the exact amount of detergent needed for cell lysis [76]. Also, DNase I concentrations may need to be adjusted empirically depending on initial type and number of cells, the lot of DNase I used and the exact purpose of the experiment [84]. Overall, DNase-seq represents a reliable and robust way to identify active regulatory elements across the genome and in any cell type from a sequenced species, without *a priori* knowledge of additional epigenetic information. Its reliability as a TF footprint detection assay in a temporal scale is questionable and needs to be investigated further in detail.

##### FAIRE-seq

One of the easiest methods for directly probing nucleosome-depleted areas of a genome is FAIRE (Formaldehyde-Assisted Isolation of Regulatory Elements) (Figure 1), although the high background in the output data limits its usefulness [15, 86–89]. FAIRE is based on the phenol-chloroform separation of nucleosome-bound and free areas of a genome in the interphase and aqueous phase respectively. The procedure involves the initial crosslinking of chromatin with formaldehyde to capture *in vivo* protein-DNA interactions, and subsequent shearing of chromatin with sonication. Following phenol-chloroform extraction, nucleosome-depleted areas of the genome are released to the aqueous phase of the solution due to much higher crosslinking efficiency of histones to DNA, compared to other regulatory factors [87, 90]. The chromatin-accessible population of fragments can then be detected by quantitative PCR, tiling DNA microarrays [15, 86] or more recently with paired-end or single-end NGS (FAIRE-seq) [87, 91].

Initially demonstrated to identify accessible regulatory elements in *Saccharomyces cerevisiae*[90], FAIRE has been extended to a wide range of eukaryotic cells and tissues, consistently demonstrating a negative relationship with nucleosome occupancy and an overlap with various cell type-specific marks of active chromatin [15, 45, 86, 87, 92, 93]. This assay has been instrumental for the identification of active regulatory elements in a number of human cell lines by ENCODE [26]. It has been used widely to detect open chromatin in normal and diseased cells [86, 91, 94, 95], to associate specific chromatin states with known sequence variants of disease susceptibility [91] or allele-specific signatures [96], and to decipher the effects of TF binding to chromatin structure [97, 98].

Overall, FAIRE enriches directly for areas of active chromatin, with the added benefit that the nucleosome-depleted regions are not degraded, it can be applied to any type of cells or tissue and that there is no requirement for initial preparation of cells and laborious enzyme titrations [15, 86, 89, 94]. FAIRE has been shown to identify additional distal regulatory elements not recovered by DNase-seq, although it remains unclear what these sites represent [94]. In addition, FAIRE overcomes the sequence-specific cleavage bias observed with MNase and DNase I, and thus represents an ancillary approach for these assays [52–54, 60, 99].

Success of any FAIRE-seq experiment heavily depends on adequate fixation efficiency that can alter depending on cell permeability, composition and a variety of other physiological factors. For most mammalian cells, 5 minutes of fixation time is usually ample [89]. Fungi and plants may require a much higher fixation time [15, 93] or improved fixation solutions [100] and optimization is necessary to avoid inconsistent results. Also, FAIRE has lower resolution in identifying open-chromatin at promoters of highly expressed genes compared to DNase-seq [94]. FAIRE’s major limitation, that far outweighs all benefits, is that it has a lower signal-to-noise ratio compared to the other chromatin accessibility assays. This high background makes computational data interpretation very difficult, with only strong recovered signal being informative.

##### ATAC-seq

ATAC-seq is the most current method for probing open chromatin, based on the ability of hyperactive Tn5 transposase [101, 102] to fragment DNA and integrate into active regulatory regions *in vivo* (Figure 1) [103]. During ATAC-seq, 500–50,000 unfixed nuclei are tagged *in vitro* with sequencing adapters by purified Tn5 transposase. Due to steric hindrance the majority of adapters are integrated into regions of accessible chromatin that are subsequently submitted to PCR for library construction followed by paired-end NGS. This method has been recently used in a eukaryotic line to uncover open chromatin, nucleosome positioning and TF footprints genome-wide [103]. Despite its limited application so far, ATAC-seq is attracting a growing interest due to its simple and fast two-step protocol, its high sensitivity with a low starting cell number (500 to 50,000 cells) and the ability to study multiple aspects of chromatin architecture simultaneously at high resolution.

The sensitivity and specificity of ATAC-seq is similar to DNase-seq data obtained from approximately three to five orders of magnitude more cells, and it diminishes only for really small numbers of cells [103]. The ATAC-seq protocol does not involve any size-selection steps and can thus identify accessible locations and nucleosome positioning simultaneously. However, its ability to map nucleosomes genome-wide is limited to regions in close proximity to accessible sites [103]. The most challenging aspect of ATAC-seq is the analysis of the sequence data, since generalized methods are unavailable or limited. With the additional demonstrated ability for analyzing a patient’s epigenome on a clinical timescale [103], we foresee ATAC-seq to become the preferred method for the study of chromatin structure in the near future.

### Chromatin accessibility high-throughput sequence data analysis

Detection of chromatin accessibility genome-wide with all the above methods requires initial library construction and use of NGS [31, 104]. Resulting data represents an average *in vivo* snapshot of chromatin accessibility, as represented in the constructed sequencing libraries. Normally, a specialized sequencing facility performs library construction and sequencing using the appropriate kits for the operated sequencer. Otherwise, a research laboratory can use in-house instrumentation and manufacturer or custom library protocols, with the latter being more cost efficient.

Although a number of sequencers are currently available for deep sequencing, most researchers use Illumina next-generation platforms due to the high number of molecules (tag count) that can be sequenced per sample. Tag count represents the most instrumental parameter of output sequencing quality. The number of tags that need to be sequenced depends on the goal of the specific experiment, with nucleosome mapping and TF footprinting experiments requiring higher coverage compared to standard chromatin accessibility detection. To obtain a target coverage depth per sample, the researcher should take into account the minimal number of mappable tags delivered by the instrument in use and adjust accordingly the number of multiplexed samples per lane of flow cell (for details read [31]). A secondary parameter of sequencing quality is tag length, which is mainly a function of the applied sequencing chemistry and currently varies between approximately 36 to 300 bp. Generally speaking, paired-end and longer-read sequencing provides the most accurate results and is recommended whenever possible, especially for areas of the genome with low-complexity or many repetitive elements [31, 104]. However, in most experimental cases chromatin accessibility can be accurately determined with single-end, shorter-length reads without the unnecessary additional expense.

**Figure 2:**
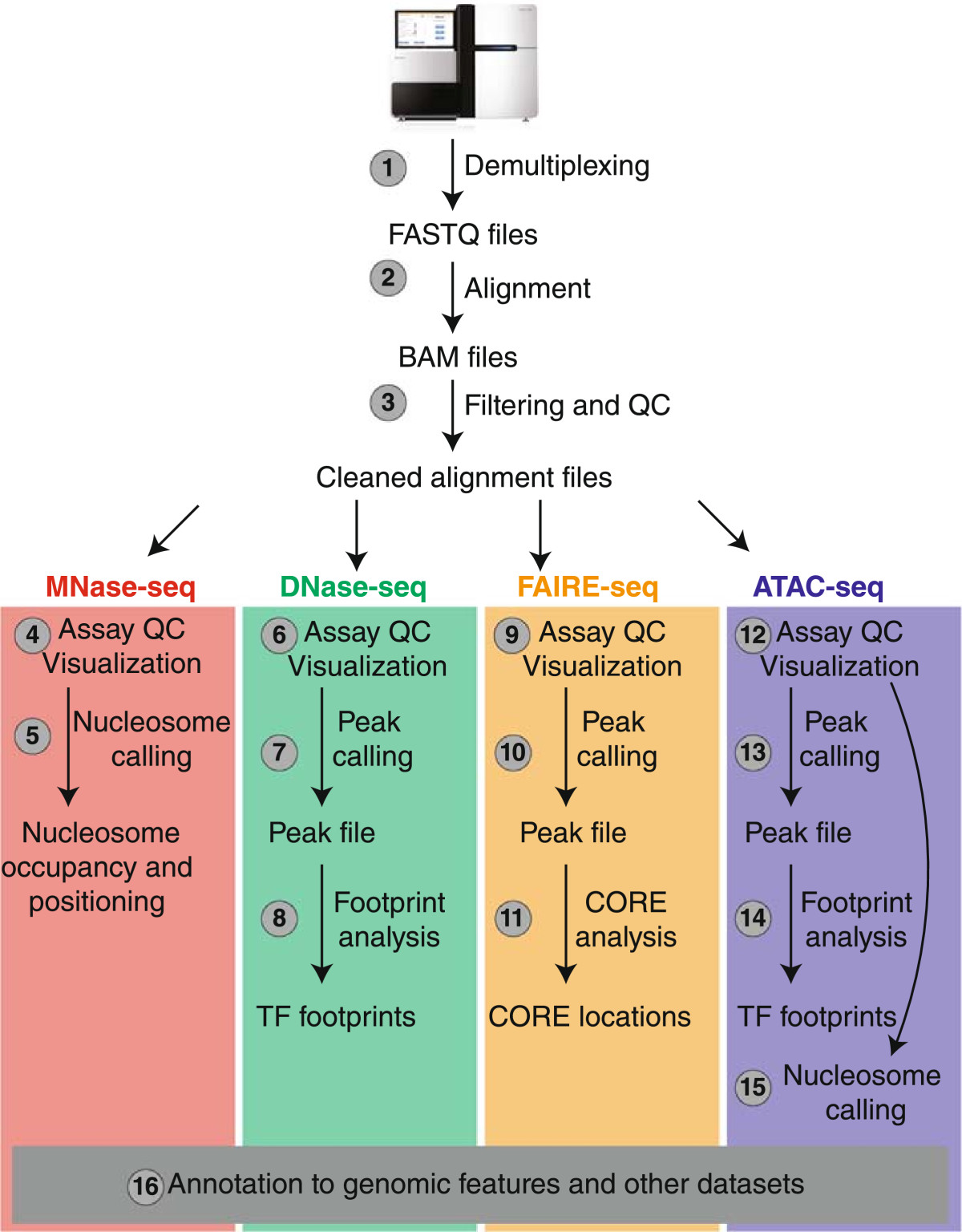
**Chromatin accessibility high-throughput data analysis workflow.** Chromatin accessibility data analysis involves a number of stages with progressively increased level of difficulty and advanced requirements for computational and genomics expertise. All major steps of analyses, from sequence tags to data annotation/integration are shown in a comprehensive workflow format (read text for additional details).

The vast amount of generated sequencing data is subsequently analyzed using a variety of analytical tools, with progressively increased level of difficulty and advanced requirements for computational and genomics expertise. As a result, data analysis along with computing power and storage capacity, are often regarded the current bottleneck in chromatin accessibility experiments. Below we discuss each stage of analysis with separate references to specific chromatin accessibility assays, and more specialized reviews whenever necessary, in an attempt to provide a comprehensive analysis workflow for the novice chromatin accessibility researcher (Figure 2 and Table 2). We mainly discuss analysis of sequence data generated with Illumina-based chemistry since this is the currently most preferred approach.

**Table 2:** **Chromatin accessibility high-throughput sequence data analysis**

	Detection of enriched regions	Estimation of nucleosome organization and TF occupancy metrics
**MNase-seq**	1. GeneTrack [126]	1. Nucleosome positioning algorithms [48, 58, 111, 144]
	2. Template filtering algorithm [58]	2. Nucleosome occupancy algorithms [48, 145]
	3. DANPOS [109]	3. V-plots for TF occupancy [50]
	4. iNPS [127]	
**DNase-seq**	1. F-Seq [129]	1. Digital genomic footprinting algorithms [19, 78, 83, 85, 128, 146–149].
	2. Hotspot, DNase2Hotspots [21, 130]	2. Nucleosome and TF occupancy algorithms [150]
	3. ZINBA [131]	3. CENTIPEDE [151]
	4. MACS [132]	
**FAIRE-seq**	1. MACS2; https://github.com/taoliu/MACS/, [132]	Not available
	2. ZINBA [131]	
**ATAC-seq**	1. ZINBA [131]	1. Digital genomic footprinting algorithms [19, 78, 83, 85, 128, 146, 149]
	2. MACS2; https://github.com/taoliu/MACS/, [132]	2. CENTIPEDE [151]
	3. Hotspot, DNase2Hotspots [21, 130]	

#### Stage 1 analysis

Overall, most initial data analysis steps are the same for all chromatin accessibility assays discussed above and are normally done by the NGS facility performing the sequencing reactions. These steps include demultiplexing, alignment to a reference genome, tag filtering and measurement of sequencing quality control (QC) (Figure 2). The goal of this stage of analysis is to determine if the sequencing was done with the required depth of coverage and to prepare BAM alignment files for downstream assay-specific analyses.

Initially raw sequencing reads are demultiplexed (Step 1) based on index information into FASTQ files with CASAVA (Illumina) and aligned (Step 2) to a user-defined reference genome (that is human, mouse, and so on) [105]. A number of aligning software is available, such as Maq, RMAP, Cloudburst, SOAP, SHRiMP, BWA and Bowtie [106]. The last two represent the most popular aligning software packages currently. During the alignment process data is filtered (Step 3) to remove overrepresented areas of the genome due to technical bias. Tag filtering is often performed with SAMtools [107] or Picard tools (http://broadinstitute.github.io/picard). For ATAC-seq data specifically, mapped fragments below 38 bp are removed since that is the minimum spacing of transposition events due to steric hindrance [102]. Also, ATAC-seq reads mapping to the mitochondrial genome are discarded as unrelated to the scope of the experiment. Sequencing performance QC (Step 3) is performed during the alignment process, by estimating specific statistical metrics (that is total number of reads, % of unpaired reads, % of reads aligned 0 times, % reads aligned exactly once, % of reads aligned more than once, and overall alignment rate) for each sequenced sample.

#### Stage 2 analysis

This is the stage where most researchers begin their analysis and includes assay QC, data visualization, and detection of genomic regions of enrichment (nucleosome or peak calling; Figure 2).

##### Assay QC and data visualization

The goal of this analysis is to determine if the experiment was successful and is often performed by constructing composite plots and by visualization (Steps 4, 6, 9, and 12). Multiple tools are available for generating composite plots including ArchTEX [108], DANPOS-profile [109], and CEAS [110]. For example, TSSs have been shown to be chromatin accessible on average across all eukaryotic genomes [48, 111, 112]. A drop of composite plot signal intensity is expected at this feature when analyzing MNase-seq data, whereas DNase-seq, FAIRE-seq and ATAC-seq data will exhibit an overall increase at the same sites. ArchTEX can also be used to assess the cross-correlation of MNase-seq data, with successful experiments exhibiting enrichment at nucleosomal banding sizes [113]. ATAC-seq QC can be further performed by estimating the percentage of sequence reads that map to the mitochondrial genome and by generating ‘insert size metric plots’ using Picard tools. High quality ATAC-seq data will coincide with a low percentage of mitochondrial reads, and a distribution of insert sizes that depicts a five to six nucleosomal array along with ten bp periodicity of insert sizes.

A number of publicly available stand-alone genome browser tools [114], including Artemis [115], EagleView [116], MapView [117], Tablet [118], Savant [119], and Apollo [120], can be used to visualize raw tag density profiles (and enriched genomic regions, see below) in relation to available annotation tracks. The University of California Santa Cruz (UCSC) [121] and the Integrative Genomics Viewer (IGV) [122] represent some of the most powerful options currently. UCSC provides a plethora of information on whole-genome and exome sequencing, epigenetic and expression data, single nucleotide polymorphisms (SNPs), repeat elements and functional information from the ENCODE and other research projects. It supports incorporation of personally generated data as BED, BedGraph, GFF, WIG and BAM files, so that a researcher can compare his/her own data directly with the publicly available one. IGV represents another efficient, high-performance and intuitive genomics visualization and exploration tool, characterized by its ability to handle large and diverse datasets on a desktop computer. The user can input a variety of data types to compare them with publicly available data from the ENCODE, Cancer Genome Atlas [123], 1000 Genomes [124] and other projects.

#### Detection of enriched regions

##### MNase-seq data

In a typical MNase-seq experiment, chromatin accessibility is probed *indirectly* by deciphering areas of the genome that are occluded by nucleosomes (Figure 1). The location of each mapped tag is identified by the genomic coordinate of the 5′ end in the forward or reverse strand and represents the strand-corresponding nucleosome border (unshifted tag) [125]. Tags can also be shifted 73 bp [111] or extended for 120 to 147 bp [48, 113] towards the 3′ direction to represent the midpoint or full nucleosome length respectively. For organisms with short linkers a 120 bp extension provides better nucleosome resolution and reduces overlaps between neighboring nucleosomes [113]. With paired-end sequencing, the nucleosome midpoint is assumed to coincide with the midpoint of the forward and reverse reads. To map consensus nucleosome positions representative of the average cell population, overlapping reads have to be clustered over genomic regions (Step 5).

Current popular nucleosome calling methods are GeneTrack [126], template filtering [58], DANPOS [109], and iNPS [127]. GeneTrack implements a Gaussian smoothing and averaging approach to convert measurements at each genomic coordinate into a continuous probabilistic landscape. Nucleosomes are then detected as the maximal data subset from all local maxima with a user-defined exclusion zone that represents the steric exclusion between neighboring nucleosomes (that is 147 bp) and is centered over each assigned peak. The template filtering algorithm was developed to control for the variable MNase cut patterns observed at different concentrations of MNase digestion. This method uses a set of templates, which match frequently found distributions of sequence tags at MNase-generated nucleosome ends, to extract information about nucleosome positions, sizes and occupancies directly from aligned sequence data. However, the current version of template filtering is only suitable for small genomes (approximately 12 MB) due to memory limitations. iNPS differs from other nucleosome callers in that it uses the wave-like structure of nucleosome datasets as part of its smoothing approach. iNPS detects nucleosomes with various shapes from the first derivative of the Gaussian smoothed profile. DANPOS differs from all above approaches in that it allows the comparison of MNase-seq datasets and identifies dynamic nucleosomes based on fuzziness change, occupancy change and position shift. In addition, DANPOS performs well in assigning nucleosomes from a single experiment, and should prove an invaluable analysis tools for deciphering underlying chromatin perturbations responsible for various disease and cellular phenotypes.

##### DNase-seq data

Scientists have traditionally applied algorithms developed for ChIP-seq, without an input DNA control, to detect enriched DHSs although peculiarities of DNase-seq data render this approach unsuitable without adjustment of default settings at minimal [128]. Currently, the most widely used peak-calling algorithms for DNase-seq data analysis are the publicly available F-Seq [129], Hotspot [130], ZINBA [131] and MACS [132–135] (Step 7). F-Seq and Hotspot represent the only tools specifically developed for handling the unique characteristics of DNase-seq data. ZINBA can be applied as a general peak-calling algorithm for many types of NGS data and MACS, although initially developed for the model-based analysis of ChIP-seq data, has been successfully used as a peak-caller for DNase-seq data in many instances [136]. All these tools are based on different algorithms, parameters and background evaluation metrics (for details read [135]).

Briefly, F-Seq [129] is a parametric density estimator of sequence tag data, developed to overcome the bin-boundary effects of histogram metrics for peak enrichment [129]. F-seq implements a smooth Gaussian kernel density estimation that takes into account the estimated center of each sequence read. F-seq has been implemented in a number of studies [17, 19, 79, 94] for the identification of chromatin accessibility and the evaluation of TF footprints in relation to ChIP-seq data [17, 19, 79, 94]. However, it requires time-consuming designing for statistical testing [137]. The Hotspot algorithm [21, 130] has been widely used by the ENCODE consortium to identify regions of chromatin accessibility and represents, to our knowledge, the only DNase-seq-specific algorithm that reports statistical significance for identified DHSs [128]. The algorithm isolates localized DHS peaks within areas of increased nuclease sensitivity (‘hotspots’). Results are evaluated with false discovery rate analysis for statistical significance, employing generation of a random dataset with the same number of reads as the analyzed dataset. The newest version of Hotspot, DNase2hotspots, merges the two-pass detection in the original algorithm into a single-pass [130].

ZINBA, is a statistical pipeline characterized by its flexibility to process recovered signals with differential characteristics [131]. Following data preprocessing, the algorithm classifies genomic regions as background, enriched or zero-inflated using a mixture regression model, without *a priori* knowledge of genomic enrichment. In turn, identified proximal enriched regions are combined within a defined distance using the broad setting, and the shape-detection algorithm is implemented to discover sharp signals within broader areas of enrichment. The advantage of ZINBA is that it can accurately identify enriched regions in the absence of an input control. In addition, the software uses *a priori* or modeled covariate information (for example G/C content) to represent signal components, which improves detection accuracy especially when the signal-to-noise ratio is low or in analysis of complex datasets (for example DNA copy number amplifications). MACS a model-based analysis algorithm with wide applicability for the analysis of ChIP-seq data [138–140], has also been effectively applied for DHS detection. The algorithm empirically models the shift size of sequence reads, and employs a Poisson distribution as a background model to capture local biases attributed to inherent differential sequencing and mapping genomic properties.

A recent comparison of the above four peak callers demonstrated that F-Seq and ZINBA have the highest and lowest sensitivity respectively [135]. F-Seq has also been shown to perform better than window-clustering approaches in a separate study [129], and its accuracy can be significantly increased by reducing the peak signal threshold setting from the default value of four to a value between 2 and 3 [135].

##### FAIRE-seq data

For FAIRE-seq data the algorithm MACS [132] has been further extended to MACS2 (https://github.com/taoliu/MACS/) and performs reliably in identifying genomic regions of open chromatin (Step 10). This application is invoked by using the command macs2 callpeak and can be combined with the options broad, broad cutoff, no model, no lambda (unless a control file is given) and shift size. The algorithm uses default peak calling (q = 0.05) and broad (q = 0.10) cutoff values, but these settings can be adjusted or converted to *P*-values empirically. Once the peak-calling cutoff is set as a *P*-value, the broad cutoff value is automatically perceived as *P* also. The shift size parameter should be set as the midpoint of the average sonication fragment length in the analyzed dataset. In addition, upon availability a matched control sample can be used as input to increase detection confidence. In this case, command line parameters should be adjusted accordingly. FAIRE enrichment can also be detected using ZINBA [131]. As mentioned above, this software improves detection accuracy when the signal-to-noise ratio is low or in complex datasets. However, for high signal-to-noise datasets it performs equally well with MACS, although it is much more computationally intensive.

Identified FAIRE-seq enriched regions residing in proximity to each other, have been traditionally merged together using BedTools [141] (for detailed instructions read [142]) to form Clusters of Open Regulatory Elements (COREs) (Step 11) [91, 94, 95]. Formation of COREs allows the identification of chromatin accessibility and gene regulation patterns that may have otherwise remained undetectable in a smaller genomic scale. COREs can be also generated from all other chromatin accessibility datasets.

##### ATAC-seq data

ATAC-sec peak calling (Step 13) can be performed also by using ZINBA [103]. Alternatively, our group has found that MACS2 and Hotspot [130] perform equally well with ZINBA at identifying accessible locations (unpublished data).

#### Stage 3 analysis

This stage of analysis involves estimation of various parameters of the epigenomic landscape, including nucleosome spacing, positioning and occupancy [31], and TF binding for footprinting experiments (Figure 2).

##### MNase-seq data

Nucleosome or translational positioning indicates the position of a population of nucleosomes in relation to DNA, and considers a specific reference nucleosome point like its start, dyad or end [143]. Translational positioning is reflected in the standard deviation of the population positioning curve, and is used to distinguish between strongly and poorly positioned nucleosomes [143]. Translational positioning can be further characterized as absolute, based on the probability of a nucleosome starting at a specific base *x*, and conditional, based on the probability of a nucleosome starting within an extended region with center base pair *x*[56]. Nucleosome occupancy on the other hand, measures density of nucleosome population and is reflected in the area under the population positioning curve [143]. Nucleosome occupancy is tightly linked to chromatin accessibility, and depends on the degree a genomic site is occupied by nucleosomes in all genomic configurations [56]. A number of methods have been applied to measure nucleosome positioning [48, 58, 111, 144] and occupancy [48, 145] from MNase-seq data based on the number of sequence reads that start at each base pair, assessed for a consensus nucleosome position or in a per base pair basis [56]. In addition high-resolution MNase-seq data generated using a modified paired-end library construction protocol can be analyzed using V-plots to detect TF binding. V-plots are two dimensional dot-plots that display each fragment’s length in the Y-axis versus the corresponding fragment midpoint position in the X-axis [50].

##### DNase-seq data

Stable binding of TFs in the vicinity of DHSs protects DNA from nuclease cleavage and generates DNase I footprints that at high-sequencing depth can unveil occupancy of TFs with long DNA residence times (for example CTCF and Rap1) [84, 85]. Thus, high-coverage DNase-seq data can be analyzed with specialized algorithms to detect long-standing TF binding (Step 8). Previously specialized algorithms developed for DGF have identified hundreds of TF binding sites at genome-wide resolution, by comparing the depth of DNase I digestion at TF binding sites to adjacent open chromatin and taking into account only raw counts of 5′ ends of sequencing tags [19, 78, 83, 128, 146–149]. However, some of these algorithms are inefficient for mammalian genomes [130] or publicly unavailable. The latest publicly available footprinting algorithm, DNase2TF, allows fast evaluation of TF occupancy in large genomes with better or comparable detection accuracy to previous algorithms [85]. However, it still suffers from detection inaccuracies stemming from transient TF DNA residence time and the inherent cutting preferences of DNase I like all currently available footprinting algorithms [85].

The recently reported modified approach DNase I-released fragment-length analysis of hypersensitivity (DNase-FLASH) [150] allows *simultaneous* probing of TF occupancy, interactions between TFs and nucleosomes and nucleosome occupancy at individual loci, similar to ATAC-seq. The method is based on the concurrent quantitative analysis of different size fragments released from DNase I digestion of genomic DNA, with microfragments (<125 bp) depicting TF occupancy, and larger fragments (126 to 185 bp) representative of nucleosomal elements.

##### ATAC-seq data

Analysis of ATAC-seq paired-end data can reveal indispensable information on nucleosome packing and positioning, patterns of nucleosome-TF spacing, and TF occupancy *simultaneously* at genome-wide resolution similar to DNase-FLASH [103]. Analysis is based on the distribution of insert lengths and the positions of insertions after Tn5 transposition within open chromatin of active regulatory elements (Step 15). For TF foot printing (Step 14) our laboratory uses CENTIPEDE [151] (see below), although other footrprinting algorithms are also available [19, 78, 83, 85, 128, 146–149]. For footprinting analysis, cleavage sites have to be adjusted four to five bp upstream or downstream due to the biophysical characteristics of Tn5 transposase, which inserts two adaptors separated by nine bp [102]. It is not known if footprinting detection with ATAC-seq data is factor-dependent or affected by Tn5 cleavage preferences.

#### Stage 4 analysis

Data annotation and integration represents the final and most informative stage of analysis and requires computational and genomics background on genomic organization and structure (Step 16). After identification of enriched regions and estimation of metrics of nucleosome organization and TF occupancy, it is often desirable to evaluate this data in light of relevant information from other experiments. For example, a researcher can evaluate the overlap or association of the sequence data with genomic features (that is promoters, introns, intergenic regions, TSSs, TTSs) and ontological entities (that is molecular functions, biological processes, cellular components, disease ontologies, and so on). For that purpose, BedTools (documentation is available at http://bedtools.readthedocs.org) and its sister PyBEDTools represent a versatile suite of utilities for a variety of comparative and exploratory operations on genomic features such as identifying overlap between two datasets, extracting unique features, and merging enriched regions using a predefined distance value [141, 142, 152]. Also the UCSC genome browser offers a suite of similar utilities specifically tailored for data file conversions (http://genome.ucsc.edu/util.html). Identified chromatin accessible locations can be compared against functional annotations with GREAT, to identify significantly enriched pathways or ontologies and direct future hypotheses [153].

One can also inspect enriched regions of interest for discovery of putative TF binding events using two approaches. The first approach is straightforward and is based on comparing sequence data against a database of known TF motifs. The second type of analysis can be computationally intensive and involves the *de novo* discovery of novel TF binding sites. A number of available software (MEME [154, 155], DREME [156], Patser (http://stormo.wustl.edu/software.html), Matrix Scan [157], LASAGNA [158], CompleteMOTIFs [159], and MatInspector (Genomatix) [160]), and TF motif databases (MatBase Genomatix; http://www.genomatix.de/online_help/help_matbase/matbase_help.html), JASPAR [161], TRANSFAC [162] and UniPROBE [163]) can arrogate TF motif identification and *de novo* discovery within enriched regions.

For DNase-seq and ATAC-seq experiments TF footprints can be analyzed with CENTIPEDE [151]. CENTIPEDE is an integrative algorithm for rapid profiling of many TFs simultaneously that combines known information on TF motifs and positional weight matrices, with DNase-seq or ATAC-seq cutting patterns in one unsupervised Bayesian mixture model. Combination of all this information with publicly available expression, DNA methylation and histone modification data can be instrumental for answering questions on epigenetic regulation and inheritance and unveiling long-range patterns of gene regulation and disease development [17, 19, 137]. Finally, multistep sequential data analysis can be generated and stored using Galaxy [164] or Cistrome [165].

## Conclusions

Each of the chromatin accessibility assays discussed here has inherent limitations in identifying regions of enrichment, based on the fragmentation method used and the involvement of any size selection steps. MNase-seq, DNase-seq and ATAC-seq are all based on the double enzymatic cleavage of DNA fragments and are sensitive to the excision-ability of a fragment. As shown in MNase-seq and ATAC-seq experiments, this sensitivity represents an issue only when mapping larger fragments (>100 bp) because the data is heavily biased by the overall nucleosome configuration at the region [55, 103]. In MNase-seq experiments, it was specifically shown that nucleosomes flanked by hypersensitive sites or long linkers are excised easier at low enzymatic concentrations and exhibit artificially higher nucleosome occupancy compared to nucleosomes without these characteristics, thus leading to biased results [55].

Functional annotation of accessible regions is factor-dependent and relies highly on the availability of accurate TF binding motifs and their relevant information content as well as the spatial and temporal interaction of TFs with DNA [84, 85]. Recent research supports that DNase I cleavage patterns are affected by the time of interaction of TF with their recognition sites, with depth of cleavage being proportional to residence time [85]. Consequently, transient TFs leave minimal or no detectable cut signatures and their binding cannot be identified with any of the current footprinting algorithms. In addition, cleavage signatures appear in genomic sites with no apparent protein binding, providing further support that footprint profiles may arise as a result of inherent DNase I cleavage bias instead of protein protection from enzymatic activity. Thus, to accurately characterize gene regulatory networks from accessibility data, we need comprehensive TF motif databases generated using *in vivo*/*in vitro* assays or computationally based *de novo* motif discovery algorithms. More importantly there is an imminent need to further investigate the applicability of DNase-seq, and ATAC-seq for that matter, to accurately detect factor-chromatin interactions in dynamic cellular settings. It is possible that future footprinting algorithms will be able to accurately identify only a subset of TF binding events based solely on analysis of footprints with high depth (above a statistically validated threshold), and not on generic analysis of all cleavage profiles.

Currently, most researchers compare their chromatin accessibility data to other published datasets. Although, this approach is advantageous when public datasets are available, it does not explain the cause of identified differences. In the absence of a ‘golden standard’, experimental and computational approaches need to be compared against independently generated data. For example, active regulatory regions identified by chromatin segmentation of histone modification ChIP-seq data, can serve as an independent control for experimental and computational accuracy of current chromatin accessibility assays. Finally, development of specialized statistically supported peak-calling algorithms for DNase-seq and ATAC-seq data will be instrumental in the identification of active regulatory elements genome-wide. We foresee that future applications of chromatin accessibility will include the detection of allele-specific effects to identify functionally important SNPs, use of accessibility in eQTL studies to link regulatory regions with disease phenotypes, and assessment of clinical samples for epigenetic biomarkers of disease.

## Authors’ information

MJB is an associate professor at the Department of Biochemistry at the State University of New York at Buffalo, a director of the WNYSTEM Stem Cell Sequencing/Epigenomics Facility, a co-director of the UB Genomics and Bioinformatics Core, and an adjunct faculty for the Cancer Genetics Roswell Park Cancer Institute and the Department of Biomedical Informatics. He has extensive experience with chromatin accessibility assays and bioinformatics analysis of related data. He is currently the head of an active laboratory focused on epigenomic profiling and the detection of key determinants for gene regulation, disease development and progression. MT is a senior research scientist/project leader in MJB’s laboratory involved in a number of studies on epigenetic regulation.

## Authors’ original submitted files for images

Below are the links to the authors’ original submitted files for images. Authors’ original file for figure 1 Authors’ original file for figure 2

## Abbreviations

ATAC: assay for transposase-accessible chromatin
bp: base pair
ChIP-seq: chromatin immunoprecipitation with deep sequencing
COREs: Clusters of Open Regulatory Elements
DGF: digital genomic footprinting
DHS: DNase I hypersensitive site
DNase I: deoxyribonuclease I
FAIRE: Formaldehyde-Assisted Isolation of Regulatory Elements
MNase: micrococcal nuclease
NGS: next-generation sequencing
PCR: polymerase chain reaction
QC: quality control
SNPs: single nucleotide polymorphisms
TF: transcription factor
TSSs: transcription start sites
UCSC: University of California Santa Cruz.
