# Supplementary figures and images for: Chromatin accessibility: a window into the genome
Source: Epigenetics Chromatin. 2014 Nov 20;7:33. doi: 10.1186/1756-8935-7-33 (PMC4253006; doi:10.1186/1756-8935-7-33)

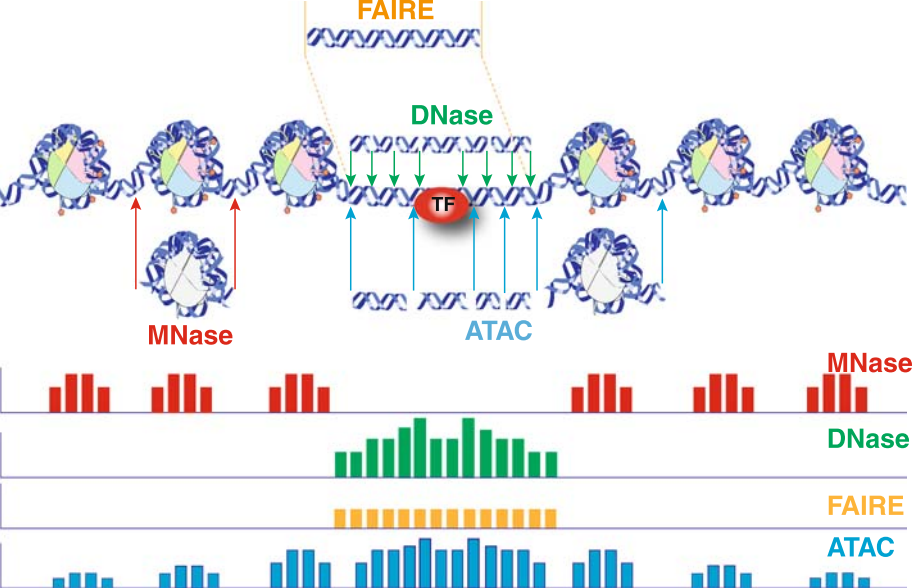

Supplement: Supplementary file 1 — Authors’ original file for figure 1 [file 13072_2014_338_MOESM1_ESM.pdf]

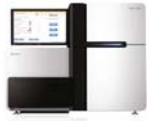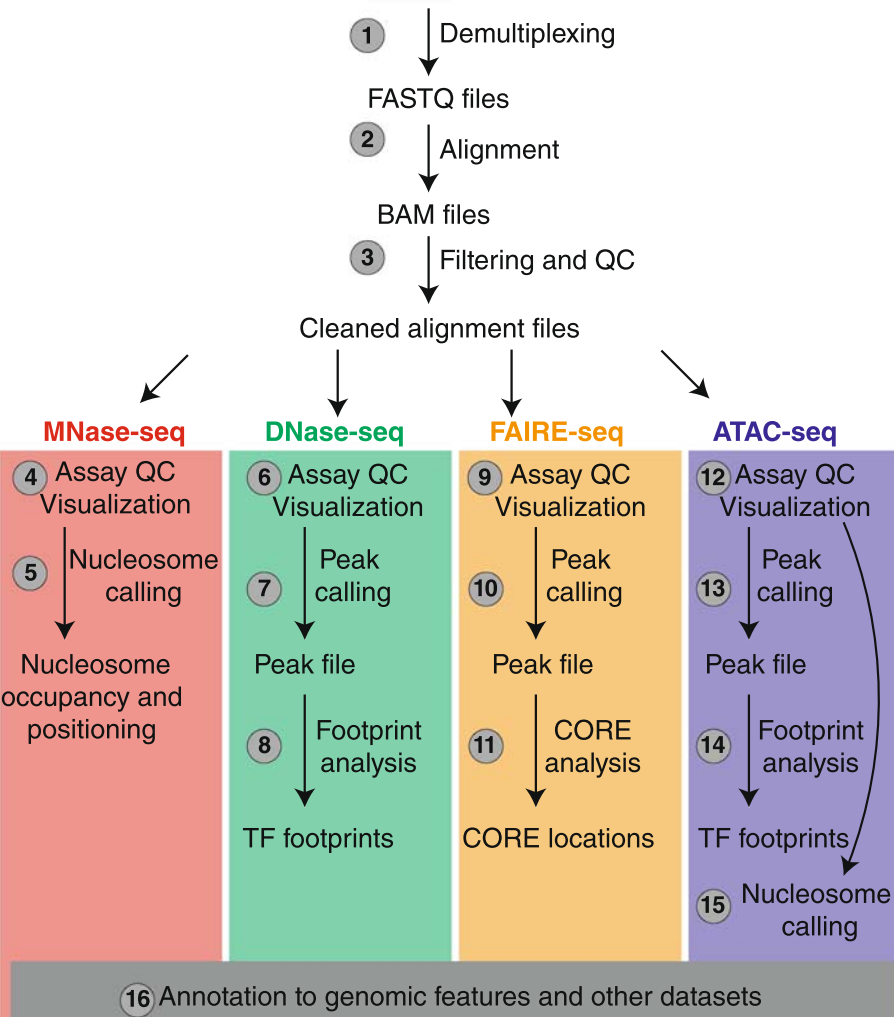

Supplement: Supplementary file 2 — Authors’ original file for figure 2 [file 13072_2014_338_MOESM2_ESM.pdf]
